# The Effects of Chinese Medicine on Activation of Wnt/*β*-Catenin Signal Pathway under High Glucose Condition

**DOI:** 10.1155/2015/295135

**Published:** 2015-10-01

**Authors:** Wei Liu, Xiaochun Liang, Dan Yang

**Affiliations:** Department of Traditional Chinese Medicine, Peking Union Medical College Hospital, Peking Union Medical College, Chinese Academy of Medical Sciences, No. 1 Shuaifuyuan, Dongcheng District, Beijing 100730, China

## Abstract

Diabetes mellitus (DM) is a metabolic disease characterized by chronic hyperglycemia and a series of complications. The Wnt/*β*-catenin signaling pathway is a complex protein interaction network, which is also a key regulator of cell proliferation and differentiation. Many scholars have found that high glucose can activate the Wnt signaling pathway. However, the effects of activation of this pathway in the presence of high glucose levels during the progression of diabetes still remained unclear. Here, we provide a review of the study on the effects of high glucose state on the Wnt/*β*-catenin signal pathway and the influence of Chinese medicine on it.

## 1. Introduction

Diabetes mellitus (DM) is a heavy burden for patients worldwide, and the number of affected individuals is growing significantly. By 2030, it is estimated that it will affect almost 552 million [1]. High glucose is the initiating factor of DM and a series of complications, the mechanisms of which are intricate and complex. The Wnt/*β*-catenin signaling pathway is a complex protein interaction network, which is also a key regulator of cell proliferation and differentiation. Many scholars have focused on its important role during embryonic development [2], the proliferation and differentiation of osteoblasts [3, 4], and tumorigenesis [5, 6]. It has also been reported that Wnt/*β*-catenin signaling can be promoted by the highly conserved gene* Fezf2* to stimulate neuronal differentiation during forebrain development [7]. Recent investigations have highlighted the role of the Wnt signaling pathway in metabolic homeostasis and its implication in diabetes as well as other metabolic diseases. It has been confirmed in recent years that high glucose can activate this pathway [8]. This paper provides a review of studies on the effects of the Wnt/*β*-catenin signal pathway in the presence of high glucose levels and the influence of Chinese medicine on it.

## 2. Wnt/***β***-Catenin Signaling Pathway

The Wnt signal pathway mainly includes the following: extracellular factor (Wnt), transmembrane receptors (frizzled), cytoplasmic protein (*β*-catenin), nuclear transcription factor (TCFS/LEF), and a series of proteins [9]. Many recent studies have shown that the Wnt/*β*-catenin signaling pathway is closely related to diabetic nephropathy [10–12], diabetic myocardium [13], and diabetic retinopathy [14, 15].

Wnts are secreted glycoproteins expressed in the developing somites and surrounding tissues that function as extracellular signals to be part of a signaling cascade in a wide group of organisms. They are a family of factors involved in the embryonic development and regulate many processes including embryonic patterning, fate specification, axon guidance, synaptogenesis, stem cell-like renewal, cell specification, proliferation, migration, adhesion, survival, differentiation, and apoptosis. Signaling regulated by Wnt ligand binding plays an important and often essential role in the processes during growth and development. According to the different modes of Wnt protein mediated signal transduction, the Wnt signal transduction pathway can be divided into the canonical Wnt signaling pathway, the noncanonical Wnt signaling pathway, the planar cell polarity (PCP), and the protein kinase A pathway [16]. It is known that there are 19 members of the Wnt protein family [11]. Feigenson et al. [17] used Wnt-3a cultured oligodendrocyte precursor cells containing medium and discovered that the myelin basic protein was associated with reduced myelin formation, thus suggesting that Wnt-3a inhibited the differentiation from oligodendrocyte precursor cells into immature oligodendrocytes through the Wnt/*β*-catenin signaling pathway.

The canonical pathway resulted in *β*-catenin accumulation through interaction between Wnt protein and cytoplasmic proteins, which interacted with transcription factors and attached to the DNA sequence of -YCTTTGWW to regulate its downstream gene transcription. The noncanonical pathway improves the levels of intracellular calcium by activating different elements of heterotrimeric G proteins to activate the Wnt/Ca2 + channels [18]. Among them, *β*-catenin is the key factor of the canonical Wnt signaling pathway, which is a cytosolic protein that is degraded by the ubiquitin proteasome system by glycogen synthase kinase 3 beta (Gsk-3*β*) phosphorylation. It forms a complex after combining with LEF/TCF and Smad4, thereby promoting the latter into the nucleus and regulating the expression of specific target genes. It is the main effector molecule cytoplasm within the classical Wnt approach and is also associated with the intercellular junctions and cadherin [19, 20]. The concentration of *β*-catenin in the cytoplasm determines the activation and inhibition of the canonical Wnt pathway [21]. Inhibition of Gsk-3*β* phosphorylation can result in *β*-catenin accumulation in the nucleus and activation of the Wnt pathway in the cytoplasm. Therefore, increased expression of *β*-catenin suggests the activation of Wnt/*β*-catenin signaling pathway, and inhibitors of Gsk-3*β* are viewed as an activator of Wnt/*β*-catenin signal pathway [22, 23].

## 3. Activation of Wnt/***β***-Catenin Signal Pathway in the Presence of High Glucose Levels

**Figure 1:**
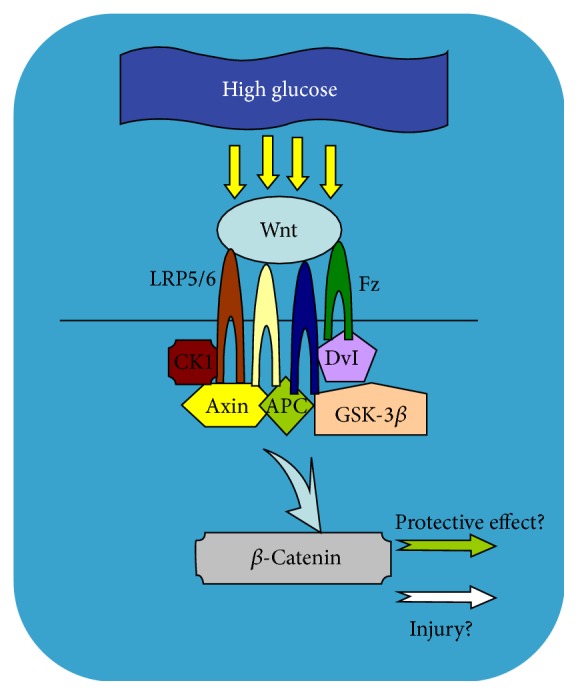
Wnt/*β*-catenin signaling pathway in high glucose condition.

Many scholars have confirmed that high glucose levels can activate the Wnt/*β*-catenin signaling pathway, but the activation of this pathway is a protective response of organization or an injury is still controversial. The possible relationship between high glucose and the activation of this pathway is shown in Figure 1. The study by Chong et al. [24] showed that the key enzyme GSK-3*β* in the Wnt/*β*-catenin signal pathway was phosphorylation inhibited at high glucose levels, and the activation pathway can inhibit the degradation of apoptosis nuclear DNA, thus playing a protective role in a variety of vascular endothelial cells. Yang et al. [25] reported that, in diabetic rat models induced by STZ, the expression of Wnt/*β*-catenin signaling pathway is upregulated, and *β*-catenin as the core of the upstream gene APC and downstream gene c-Myc was expressed and upregulated in the islet regeneration process to promote the regeneration of damaged pancreatic islet cell. Sun et al. [26] used a special puncher to create a round hole on both sides of the middle of the type 1 diabetic rat dorsal spine to create skin defects under aseptic conditions. Then, they divided the rats into diabetes, lithium chloride (pathway activator), and epidermal growth factor groups to observe the wound healing and to detect the expression of *β*-catenin. The results showed that the activation of Wnt/*β*-catenin signaling pathway can promote the healing of diabetic wounds. Some scholars showed that in mouse podocytes early diabetic podocyte injury was caused by upregulation of transient receptor potential cation channel 6 (TRPC6), which is regulated by the canonical Wnt signalling pathway. This indicates that the Wnt/*β*-catenin signalling pathway may potentially be active in pathogenesis of TRPC6-mediated diabetic podocyte injury [27].

On the other hand, Huo et al. [28] used different concentrations of glucose on rat peritoneal mesothelial cells and immunohistochemistry to detect the *β*-catenin protein expression on cells. They also used RT-PCR assay for the detection of *β*-catenin mRNA. The results showed that high glucose can induce the increased expression of *β*-catenin in rat peritoneal mesothelial cells, indicating that high glucose levels can upregulate the expression of *β*-catenin induced peritoneal injury leading to fibrosis. The research of Hwang et al. [11] and Rooney et al. [29] showed that Wnt/*β*-catenin signal pathway was closely related to the occurrence of diabetic nephropathy renal interstitial fibrosis and that blocking or inhibiting this pathway might be a new target for treatment of diabetic nephropathy. Yan et al. [30] reported that, in the process of diabetic nephropathy, activation of the canonical Wnt pathway may be involved in the high glucose mediated transdifferentiation process of renal tubular epithelial cells leading to renal interstitial fibrosis. Liu and Lai [31] cultured SD rat glomerular podocytes in different glucose concentrations, detecting nephrin and activation of *β*-catenin and Wnt-1 expression by indirect immunofluorescence and Western blot analysis, and results showed that, after 12 h of high glucose exposure, podocyte Wnt-1 and activation of *β*-catenin expression began to increase and reached its peak at 24 h. The Wnt/*β*-catenin signaling pathway may be involved in the phenotypic transformation of podocytes induced by high glucose levels. García-Jiménez et al. [32] reported that the enhanced expression of the Wnt/*β*-catenin signal pathway in tumor cells at high glucose levels may be one reason for certain cancers in DM patients. Li et al. [14] set up the DM rat model induced by STZ, with Evans blue detection of retinal vascular permeability, immunohistochemistry, and Western blot analysis for detection of the rat retina and trypsin digested retinal microvascular *β*-catenin protein. Results showed that, after 12 W, the expression of retinal and microvascular *β*-catenin protein increased significantly, and the extent of this increase is associated with the duration of DM, thereby suggesting that the occurrence of *β*-catenin protein may be involved in early diabetic retinopathy. However, the research was only limited to the detection of *β*-catenin protein and still could not fully explain the role of Wnt/*β*-catenin pathway. Portal-Núñez et al. [33] also observed the STZ induced DM rat models with the parathyroid treatment group as the control, using the gene chip technology and rt-PCR analysis of Wnt/*β*-catenin signal pathway change and analysis of *β*-catenin protein expression by immunohistochemical method change. The results showed that *β*-catenin expression in both osteoblasts and bone cells in DM rats decreased significantly, which was not due to a decrease in bone cell activity. In addition, they also found that, in the osteoblast related *β*-catenin signal transduction process, transfer of *β*-catenin to the nucleus was significantly reduced, thus leading to failure of intracellular signal transduction. It proved that dysregulation of the Wnt/*β*-catenin pathway was involved in the occurrence and development of DM osteoporosis. Wang et al. [34] detected the expression of *β*-catenin and its downstream target gene WISP-1 in Wnt signal pathway in STZ induced DM rats myocardial tissue by immunohistochemical method. The results showed that both increased, thereby suggesting that the activation of the pathway was involved in DM induced myocardial injury. There were also reports that the protein and mRNA levels of Wnt2, *β*-catenin, and c-Myc were progressively increased 4, 8, and 12 weeks following DM. However, the expression of the endogenous Wnt inhibitor Dickkopf-1 was increased after STZ injection and then decreased as diabetic cardiomyopathy developed. Jia et al. [9] found that the expression of Wnt and cytoplasmic *β*-catenin was upregulated in proximal tubular epithelial cells under DN conditions both in vitro and in vivo. Injection of LRP5 and LRP6 antibodies suppressed activation of the Wnt pathway and decreased the formation of extracellular matrix in DN animal models, suggesting that Wnt/*β*-catenin signaling might be involved in tubular-interstitial fibrosis in DN. It was also reported that Wnt/*β*-catenin/GSK3*β* signaling pathway is activated in the development of diabetic cardiomyopathy [35]. Qi et al. [36] found that lithium chloride-induced Wnt signaling activation downstream of the pigment epithelium-derived factor (PEDF) interaction site attenuated the inhibitory effect of PEDF and rescued the wound-healing deficiency in diabetic mice. These results suggest that elevated circulating PEDF levels contribute to impaired wound healing in the process of angiogenesis and vasculogenesis through the inhibition of Wnt/*β*-catenin signaling. In addition, some researchers have found that enhanced proliferation, accompanied by increased aerobic glycolysis, was detected in colorectal epithelium of patients with diabetes. *β*-Catenin accumulation with altered phosphorylation correlated with the proliferative changes [37].

Besides, as is known to all, T2DM is often associated with atherosclerosis. Nowadays, a lot of data have demonstrated that *β*-catenin activation is a key component of arteriosclerotic physiology, particularly in diabetic arteriosclerosis [38]. Gaudio et al. [39] found that an established modulator of the canonical Wnt signalling named sclerostin may protect against progression of vascular complications in diabetic patients, possibly by attenuating upregulation of *β*-catenin activity in the vascular cells.

Mechanisms of activated Wnt/*β*-catenin signaling in DM are very complex and still unclear now. At present, researches about the effects of this signaling pathway on DM and a series of complications were focused on the activity of the Wnt/*β*-catenin signaling pathway involved in the regulation of morphological changes and pathogenesis in cells, its effect on high glucose induced cells apoptosis via the promotion of caspase-3 and poly (ADP-ribose) polymerase cleavage, its influence on diabetic wound healing, and its effect on the regulation of bone and vascular and other metabolic processes. It has been suggested that sustained Wnt/*β*-catenin expression is essential for its protective role against cellular damage, while abnormal activation of Wnt/*β*-catenin results in adverse effects and promotes the progression of DM.

## 4. Effects of Chinese Medicine on the Pathway

Chinese medicine has multitarget and multiangle effects, which has caused wide attention. Lv et al. [40] used the high-sugar levels combined with different concentrations of Rhein in serum-free medium cultured human mesangial cells in vitro and analyzed cell proliferation by MTT method and the expression of Wnt/*β*-catenin gene in glomerular mesangial cells and the effects of Rhein on it by RT-PCR method. The results showed that, in the basic state, mesangial cells express a certain amount of Wnt/*β*-catenin. After glucose stimulation, the expression of the Wnt/*β*-catenin gene was increased, thereby suggesting that Rhein may inhibit the high glucose induced proliferation of mesangial cells by downregulation of Wnt/*β*-catenin gene expression. Huang et al. [41] divided the human proximal tubular epithelial cells into normal glucose group, high glucose group, and high glucose + tanshinone IIA intervention group. Immunohistochemistry and Western blot analysis were used to observe the protein expression of *β*-catenin, epithelial cell marker protein of E-cadherin, and mesenchymal cell marker protein *α*-SMA, and RT-PCR was used to detect the mRNA expression of *β*-catenin, epithelial cell marker protein of E-cadherin. The results showed that the final concentration of 100 *μ*mol/L tanshinone IIA can be significantly reduced by the ectopic expression of *β*-catenin, and the expression of *β*-catenin in nuclear protein and mRNA decreased significantly at this concentration, thereby indicating that the Wnt/*β*-catenin signaling pathway is involved in the high glucose induced transdifferentiation of renal tubular epithelial cells. Tanshinone IIA could inhibit this process by downregulating the expression of the Wnt/*β*-catenin signaling pathway activity and protecting the kidneys. Deng and Fang [42] randomly divided STZ rats into the diabetic nephropathy model group, the* Astragalus* group, the diabetic nephropathy model group + losartan treatment group, and the diabetic nephropathy model group +* Astragalus* combined with losartan treatment group. Immunohistochemistry and FQ-PCR methods were used to detect protein expression as well as expression of Wnt4, *β*-catenin, and TGF-*β*1 mRNA in renal interstitium. The results showed that* Astragalus* can protect the kidney by downregulating the expression of Wnt4, *β*-catenin, and TGF-*β*1 in renal interstitium. Duan et al. [43] observed the effects of the Yishen Capsule (mainly composed of* Astragalus*,* Angelica*, Gorgon fruit, oriental water plantain rhizome,* Rhodiola*, etc.) on diabetic nephropathy rats induced by STZ. They found that, at the end of 12 W, the expression of Wnt pathway inhibition factor-secreted frizzled related protein-1 in renal tubular-interstital cells increased compared with the control group, and *β*-catenin expression appeared in the cytoplasm or (and) nucleus. PCR results showed increases in the two mRNA expression levels. After Yishen Capsule treatment, the secreted frizzled related protein-1 in tubulointerstital cells further increased compared to the control, while *β*-catenin mRNA expression was reduced, which indicated that the Yishen Capsule could upregulate the Wnt pathway inhibiting factor, which played a role in renal protection in diabetic nephropathy rats.

In addition, the study by Lange et al. [44] found that the Wnt/*β*-catenin signaling pathway was closely related to the neural stem cell proliferation and differentiation. There were also reports showing that dysregulation of the Wnt/*β*-catenin signaling pathway may be responsible for inefficient myelin repair after human nervous system lesions [45]. Osakada et al. [46] found that the Wnt signaling pathway could promote the regeneration in the retina of adult mammals through animal experiments. Meanwhile, scholars observing the differentiation of nerve stem cells with *β*-catenin siRNA found that *β*-catenin played a key role in promoting the differentiation from neural stem cells into neurons in high-pressure oxygen in vitro [47]. Scholars explored the effect of total glucosides of peony (TGP) on Wnt/*β*-catenin signal transduction pathway expression in kidney of diabetic rats. They found that Wnt-1 and *β*-catenin expression increased in kidney of high-fat high-sugar induced type 2 diabetic rats. Compared with diabetic group, the level of serum creatinine, blood urea nitrogen, 24 h urine protein, mean glomerular area, and mean glomerular volume were decreased, renal histopathology was improved, and expression of Wnt-1 and *β*-catenin mRNA and protein was reduced in TGP group. These results showed Wnt/*β*-catenin abnormal activation in kidney of type 2 diabetic rats; TGP can improve kidney damage in diabetic rats and delay the development of diabetic nephropathy by inhibiting the Wnt/*β*-catenin signaling pathway [48].

## 5. Summary and Prospect

With the improvement of living standards, DM as a metabolic syndrome has a significant influence in the global scope, not to mention its peculiar set of complications. Many scholars have been exploring its pathogenesis, but that does not encompass the whole picture. The Wnt/*β*-catenin signaling pathway as a protein interaction network can be activated by high glucose levels. However, the relationship between the pathway and DM as well as the appearance of its complications is still unclear and needs future investigation to better clarify the accurate role. Currently, the study of the activated Wnt/*β*-catenin signaling pathway at high glucose levels is relatively more concentrated in animal experiments in vitro, and most of these experimental methods are used to detect the expression of the pathway's upstream or downstream protein and mRNA. Some parts of the comprehensive and systemic study of the pathway are still poorly understood. Regulation of specific genes on this pathway and the exact mechanism are also unclear. In addition, studies of the activation of this pathway under high glucose conditions are mainly focused on diabetic nephropathy, diabetic retinopathy, diabetic osteoporosis, diabetic cardiomyopathy, and so on. However, the effects of this pathway on diabetic peripheral neuropathy are still unknown, thereby suggesting that there are still parts of the activation of the Wnt/*β*-catenin pathway at high glucose levels that are worth exploring. Further investigation into the role of Wnt signaling during DM will functionally find novel therapeutic target for DM.

At the same time, there are few studies on the effects of traditional Chinese medicine on regulating this pathway at high glucose levels. We look forward to these studies, as long as they reveal the mechanism of diabetes and its complications from a new angle, make full use of the multidirection, multitarget role of Chinese medicine, and explore the exact effective components from the numerous complexes. The goal is to develop more effective new measures and methods for treatment of diabetes and its complications as well as its prevention so that we can relieve the physical and mental suffering of diabetic patients.
